# The Influence of Perforated Prosthetic Liners on Residual Limb Wound Healing: a Case Report

**DOI:** 10.33137/cpoj.v2i1.32723

**Published:** 2019-08-01

**Authors:** M. McGrath, J. McCarthy, A. Gallego, A. Kercher, S. Zahedi, D. Moser

**Affiliations:** 1 Blatchford Group, Unit D Antura, Bond Close, Basingstoke, RG24 8PZ, UK.; 2 Endolite North America, Miamisburg, OH, USA.

**Keywords:** Perforated Liner, Residual Limb, Wound Healing, Sweating, Vacuum, Lower Limb Prosthetics

## Abstract

**CASE DESCRIPTION:**

Good residual limb skin health is vital to successful prosthetic prescription. Unnatural loading profiles and excessive sweating can lead to skin and soft tissue problems. Perforated liners allow the transport of moisture away from the skin and allow negative pressure (a condition that has been shown to aid wound healing) to act directly on the residuum surface.

**AIM:**

Assess the effects of perforated prosthetic liner use, particularly with respect to wound healing.

**METHOD:**

Three patient histories were retrospectively reviewed following prescription of perforated prosthetic liners due to excessive sweating or prolonged residual limb health concerns. Photographic records from patient files were used to document changes in residual limb condition. Patients also provided subjective feedback regarding their experiences.

**FINDINGS:**

Two cases described active amputees with persistent blistering irritated during exercise. Another case described a patient of low mobility level with a history of residual limb skin infections. All saw their conditions heal and reported a reduction in problematic sweating. Two patients reported cancelling surgical interventions after substantial improvements with the perforated liner.

**DISCUSSION:**

These findings provide evidence that the use of perforated prosthetic liners allow improvements in residual limb health, while still permitting prosthetic use.

## INTRODUCTION

The interface between the residual limb and the prosthetic socket is, arguably, the most crucial part of successful prosthetic prescription.^1^ Without a comfortably fitting socket, the patient will not wear their prosthesis.

The skin and soft tissue of the residual limb are particularly susceptible to damage. Contrary to historic biomechanical assumptions, there is evidence to suggest that this interface should be considered as an extra joint in the lower limb, with translation and rotation,^2,3^ which lead to unnatural loading profiles. There may also be scar tissue and, if the amputation aetiology was dysvascular, the tissue is at greater risk of pressure ulcers,^4^ which cannot heal as well^5^ and could result in revision surgery or reamputation.^6^

Another issue that exacerbates the problem is excessive sweating.^1,7–10^ When questioned about the factors affecting their quality of life and their satisfaction with their prostheses, up 70% of lower limb amputees have reported that they consider sweating a problem^7,11^ and up to two-thirds claim that sweating adversely affects their activities of daily living (ADL).^12^

The causes of excessive sweating in amputees are clear. It has been reported that trans-tibial amputees use 10-40% more energy than able-bodied people to walk and perform daily tasks.^13,14^ They also have a reduced surface area (approximately 10-15% less), which affects the capacity to transfer heat energy and cool down.^15^ Consequently, the body’s natural response is to produce more perspiration. Furthermore, the use of prosthetic liners, made of silicone, polyurethane or TPE gel, creates an even warmer environment locally, around the residual limb because they often have poor thermal conductivity.^16,17^ This impermeable^18^ micro-climate is moist, warm and nutrientrich, making it ideal for bacterial growth. The sweat is stasis on the residuum surface instead of evaporating, which can lead to skin problems, such as dermatitis.^19–21^

There is evidence that liner material selection can reduce residuum temperatures,^22^ such as the Alpha SmartTemp liner (WillowWood, Roseburg, OR, USA), which uses Phase Change Material that stores and releases heat energy. However, it is unknown whether this is sufficient to reduce thermal discomfort.^23^ Regardless, even if sweating is reduced, what perspiration does occur will remain on the skin, so associated problems remain. In light of this, “breathable” prosthetic liners have been developed, which have permeable surfaces to allow the transfer of air and moisture away from the skin, such as Silcare Breathe (Blatchford, Basingstoke, UK) with laser-drilled perforations and SoftSkin Air (Uniprox, Zeulenroda-Triebes, Germany) with micro-pores. This report describes cases of patients with residual limb health conditions who were fitted with perforated liners.

## METHODOLOGY

### Participants

The case histories in this study were collected retrospectively. Once relevant cases were identified, the patients were approached and they were asked to provide written consent for their case to be described. The inclusion criteria included being over the age of 18, being able to provide informed consent, having a trans-tibial amputation, being a prosthetic limb wearer, having a history of residual limb health issues and having changed to a perforated prosthetic liner.

### Perforated liners

There were two types of perforated prosthetic liner used in these cases; one (Silcare Breathe Cushion,^a^ Blatchford, UK) used with elevated vacuum suspension (EVS) or a passive vacuum and the other (Silcare Breathe Locking,^b^ Blatchford, UK) used with a pin-lock. The ‘cushion’ version has a rounded distal cap and is used in conjunction with a suspension sleeve. A distal one-way valve in the socket allows the use of suction suspension or EVS. Laser-drilled perforations are distributed along the length of the liner. There are also perforations in the distal cap. On the pin-lock version, the perforations stop a short distance from the distal end. At the distal cap, a valve opens when the wearer bears load and closes again when the limb is lifted from the ground, creating a small area of passive vacuum in the area distal to the perforations in the wall of the liner, which facilitates the retention of the residuum within the liner.

### Data collected

Demographic data were collected from the patients in each case. These included gender, age, K level and any relevant comorbidities. Their prosthetists were asked to describe the patients’ prosthetic prescription, including suspension and ankle/foot technologies, before and after they were fitted with the perforated liner, to identify any potentially compounding factors for consideration.

Photographic evidence of residual limb health conditions was gathered to verify the clinicians’ own patient notes. Since the analysis was performed retrospectively, photos were only available when the clinician, or the amputee themselves, had seen fit to take one. These images were used for a qualitative examination of changes in residual limb health.

## FINDINGS

Three case histories were collected, covering different demographics, prosthetic preferences and residual limb health issues. Since the analysis was retrospective and gathered from different centres, the detail included in the patient records was variable between cases (**Table 1**).

### Case #1

The patient was a 41-year-old male with a right-sided, trans-tibial amputation caused by a road traffic collision approximately four years prior. He was 90kg in mass, with a body mass index (BMI) of 28.4 and he had been classified as a K3-K4 level walker.

**Figure 1:**
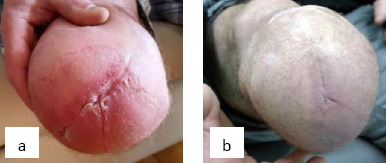
The condition of the Case #1 patient’s residual limb (a) before being fitted with a perforated liner and (b) after three months of use.

Following limb loss, the patient wished to return to his previously active lifestyle, including regular jogging, walking and cycling. However, exercise, combined with his silicone liner had led to excessive perspiration building up, remaining on the surface of his residuum and collecting distally. The patient reported that this caused relative movement between the residuum and the liner and chaffing. Blisters would develop in and around the scars at the distal end of his residuum, where sweat collected (**Figure 1a**). Jogging on consecutive days led to prosthetic disuse on the third day because the blisters made limb wearing too painful.

**Table 1:** Summary table of the cases described

Case	Age (years)	Mass (kg)	BMI	K level	Amputation type/side	Residual limb issue	Previous prescription	New prescription
#1	41	90	28.4	K3-K4	Trans-tibial /Right	Distal blisters around scarring	Cushion silicone liner with suction suspension	Pin-lock perforated liner
#2	45	100	30.2	K3-K4	Trans-tibial /Right	Posterior ulcer/wound	Pin-lock silicone liner with BladeXT foot	1st: Pin-lock perforated liner
								2nd: Cushion perforated liner with suction suspension
#3	50	106	31.0	K2-low K3	Trans-tibial /Right	Distal maceration and infection	Cushion silicone liner with suction suspension and Tres foot	Cushion perforated liner with EVS and K2-specific hydraulic ankle

In fact, the patient had booked a surgery to revise the scarring at the distal end of his residuum, in the hope that it would help to reduce blister formation. As a result of sweating issues, the patient was fitted with a locking, perforated liner on 19^th^ June 2017. It was believed that he would prefer the pin-lock version because it removed the necessity to wear a suspension sleeve. At initial fitting, the patient reported finding the liner comfortable to wear and easy to don.

A follow-up appointment was carried out after a month on 24^th^ July 2017. The patient reported that the liner remained comfortable, with good prosthetic control. He had continued jogging and walking and although he felt that his limb felt about the same temperature, there had been considerably less sweat on his limb after doffing the liner, which he described as only a “slight glisten” on the skin. His prosthetist reported no skin breakdown and improvements in the existing blistering had led the patient to postpone his surgery. There was a review appointment after three months in September 2017, at which time the patient was regularly going for 8km jogs, the blistering had healed (**Figure 1b**) and he had cancelled the surgery.

### Case #2

The patient was a 45-year-old male with a right-sided, traumatic, trans-tibial amputation. He was 100kg in mass, with a BMI of 30.2 and he had been classified as a K3-K4 level walker.

**Figure 2:**
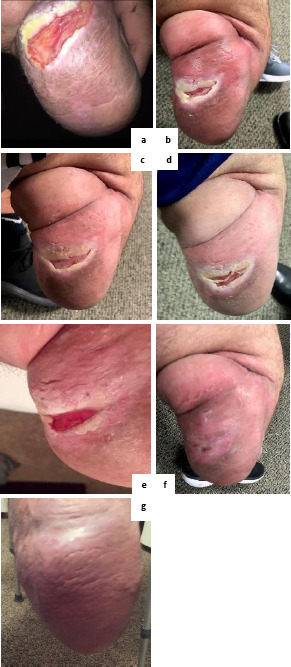
The condition of the Case #2 patient’s residual limb (a) approximately one year before being fitted with a perforated liner, (b) at the point of fitting, and after (c) 4 weeks of use, (d) 7 weeks of use (the point of changing to passive vacuum suspension), (e) 9 weeks of use, (f) 11 weeks of use and (g) 13 weeks of use.

Prior to and since his amputation, the patient enjoyed competing in motocross endurance races. For these competitions, he would wear a carbon blade-style prosthesis with a sole plate and pin-lock silicone liner. However, he struggled with excessive sweating on his residual limb and had skin issues since 2014. An ulcer developed on the posterior-distal aspect of his residuum (**Figure 2a**) and it worsened to the extent that his doctor had mentioned the possibility of further amputation to a trans-femoral level. The ulcer had persisted for over a year before he was initially fitted with a locking, perforated liner on 27th August 2018 (**Figure 2b**). In that time the patient used “no medicine, no cream, no lotion” and was advised to use “only soap and water” to clean the wound. When asked what the dermatology clinic advised him, the patient explained “they said the white heavy skin that look like a callous around the wound (**Figure 2b**), was from moisture. They told me I would have to take my leg off for 3 to 5 months for it to completely heal”, which was impractical for him as he was in full-time employment.

The residual limb condition was monitored at regular intervals over the first three months of perforated liner use at 4, 7, 9, 11 and 13 weeks. When compared to the initial fitting stage (**Figure 2b**), at 4 weeks (24th September 2018 - **Figure 2c**), the ulcer had visibly reduced in size and the wound was no longer suppurating. At 7 weeks (15th October 2018 - **Figure 2d**), Tissue was granulating and the affected area/ulcer had reduced in size. It was at this point that the patient changed to a cushion, perforated liner and Northene socket, copolymer polypropylene frame, passive vacuum adaptive expulsion valve system. Over the following 6 weeks, the ulcer reduced in size considerably (1^st^ and 12^th^ November 2018 - **Figure 2e and f**), before being considered fully healed 13 weeks after initial fitting (28^th^ November 2018 - **Figure 2g**).

Since healing, the patient has been fitted with a hydraulic ankle unit for use with his passive vacuum system. He has also continued to compete in motocross endurance events (for which he uses the locking perforated liner), achieving ‘top 5’ finishes against able-bodied competitors.

### Case #3

The patient was a 50-year-old male (mass: 106kg, BMI: 31.0), with a right-sided, trans-tibial amputation, classified as a K2 to low K3 level walker. He habitually wore an energy-storing-and-return (ESAR) foot.

**Figure 3:**
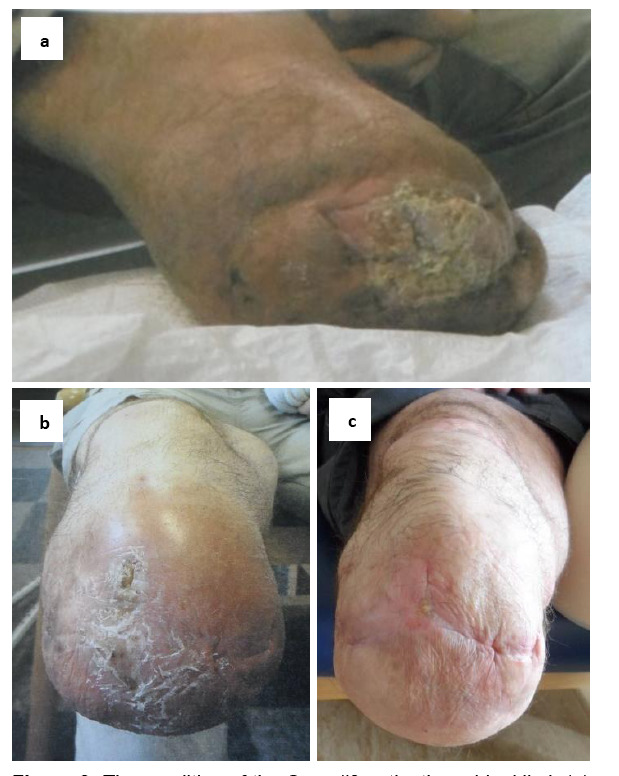
The condition of the Case #3 patient’s residual limb (a) in its worst condition in 2011, (b) in 2013 and (c) in March 2019, after continued use of a perforated liner (since 2016) with elevated vacuum suspension and hydraulic ankle (since August 2017).

The patient had chronic residual limb skin issues for approximately eight years. Excessive perspiration had led to the skin becoming macerated and infected (Figure 3a and b). The patient had reported “being on the brink of revision surgery” to remove the affected skin.

He was fitted with a perforated cushion liner, in conjunction with a passive vacuum, in 2016. In 2017 this was upgraded to an EVS system that used the movement of a hydraulic ankle unit to draw greater vacuum levels. After three months of using this prosthetic prescription, the patient reported that he thought it was “doing a great job” of keeping his skin dry; he was wound free and had had no residuum problems. The patient has continued to use this prescription for over a year, during which time his residuum remains in good health (**Figure 3c**).

## DISCUSSION

This research illustrates the health benefits of maintaining a dry residuum/socket interface by the use of perforated liners. While the use of silicone liners is primarily for comfort and impact absorption, providing a close fit and suspension,^10^ they can create warm, moist environments in which bacteria can thrive. Creating a dry environment mitigates against the risks of skin maceration and infection. Such conditions lead to uncomfortable socket fits and prosthetic abandonment. Also, intuitively, the presence of sweat will lubricate this interface, increasing relative motion between the residuum and the liner. This may affect prosthetic control and/or suspension, potentially reducing swing clearance and become a tripping hazard. Subsequent compensatory movements to improve clearance increase energy expenditure^24^ and may have a degenerative effect on musculoskeletal health.^25–27^

In their review of prosthesis thermal discomfort in 2014, Ghoseiri and Safari^10^ described available options for management of excessive heat and perspiration in the prosthetic socket. They refer to antiperspirants, local ointments and topical sprays to inhibit the physiological process of sweating but cite unpleasant odors and potential allergies as drawbacks.^10^ An alternative is the use of Botulinum Toxin (Botox) injections but this is described as invasive, requiring repeated treatment to maintain effectiveness and may potentially cause pain and/or side effects^10^ They ultimately concluded that prosthesis thermal discomfort was still an unresolved problem. Notably, this review was published prior to the commercialisation of perforated prosthetic liners.

The wounds arising from sweating can also have a substantial impact on the economics of healthcare. Treatment and care of patients with skin health conditions, such as pressure ulcers and chronic wounds, cost the UK National Health Service (NHS) up to £3.1 billion per annum.^28,29^ Although this number is for the population as a whole, amputees will be disproportionately affected, particularly those with vascular comorbidities. These issues can lead to socket adjustments or replacements, which studies have shown constitute a sizeable proportion of clinical appointments,^30,31^ creating a time and financial burden for limb centres. Additionally, two cases in this analysis reported considering surgical interventions as a result of their skin conditions, which were cancelled after tissue improvements following the use of perforated liners. Other reported treatments to reduce excessive sweating, such as Botox injections^32^ or daily antiperspirant use,^32^ can be expensive, inconvenient, produce side effects or have limited effectiveness. If a change in prosthetic liner prescription can provide the desired effect, it may be the most cost-effective approach.

As far as the authors are aware, this is the first published evidence for the effects of “breathable” liners. Some practitioners may be concerned that the perforations in liners may create areas of stress concentration that may increase the rate of deterioration of the liner itself or damage the surrounding skin. Many liner manufacturers offer warranty periods of between six and 12 months, or three months for suspension sleeves. Perforated liners have a six month warranty so although there may be a greater risk of degradation than a non-perforated liner, the longevity is still within typical industry standards for this type of prosthetic component. Although none of these three cases reported any skin issues around the perforations, the manufacturer’s Instructions For Use^c^ warn that “enlarged perforations can trap the skin and cause blisters” and recommend that should the perforations become damaged, the patient should cease the use of the liner.

It should be noted that there might have been other influences present in these cases. Although Cases #1 and #3 didn’t report the use of other wound healing treatments, such as creams or ointments, it is possible that the patients might have used these therapies without reporting such to a member of their treatment team. Han and Ceilley’s^33^ review of chronic wounds describes many topical therapies and dressings that could assist wound healing but the practicality of these treatments within a prosthetic socket environment is unclear. One of the patients also began using EVS with their perforated liner. EVS has previously been shown to reduce residuum volume fluctuation^34–38^ and relative movement,^34,37,39,40^ which reduces interface pressure.^41^ This, combined with encouraging healthier, more hydrated, more oxygenated tissue,^42^ might explain why residual limb wounds have been observed to heal faster with EVS,^43^ without discontinuing prosthetic use.^44–46^ Negative pressure wound therapy (NPWT) is a widely used technique to aid wound healing^47,48^ and has been demonstrated to be effective even for diabetic amputees.^49^ The perforations in the liner allowed the vacuum to be applied directly to the wound surface, potentially further helping healing.

Two of the cases began using hydraulic ankles with their perforated liners. These devices significantly reduce interface pressures, loading rates and deep tissue trauma,^50^ which may also have contributed to improvements in residual limb condition.

The current study was also limited in that the analysis was performed retrospectively, each clinical team had their own methods of monitoring the patients’ skin conditions and there were other prescription changes that may have influenced wound healing (e.g. prosthetic suspension method). Future work will consist of a more regimented, scientifically rigorous analysis with a wider cohort of patients. Patients will be divided into two groups; the control group will use regular silicone liners and the intervention group will use perforated liners. Patients will be monitored at pre-defined, regular intervals. The progress of wound healing will be quantified, using metrics such as wound surface area, as described in Hoskins’ work.^46^ Compounding factors, such as prosthetic componentry and suspension method will be controlled to isolate the effects of liner perforations only.

## DECLARATION OF CONFLICTING INTERESTS

The authors are full time employees of the manufacturer of the prosthetic liners evaluated in this study.

## SOURCES OF SUPPORT

Blatchford Clinical Services who documented and provided patient care.

## AUTHOR CONTRIBUTION

- **Michael McGrath**,
  Conceptualization, case collation, writing original, review and editing.
- **Joseph McCarthy**,
  Conceptualization, case collection, analysis, writing original, review and editing.
- **Ana Gallego**,
  Analysis, writing original, review and editing.
- **Alan Kercher**,
  Conceptualization, case collection, analysis, review and editing.
- **Saeed Zahedi**,
  Analysis, writing original, review and editing.
- **David Moser**,
  Analysis, writing original, review and editing.

## MANUFACTURERS’ DOCUMENTATION

^a^ https://www.blatchford.co.uk/endolite/silcare-breathe-cushion-liner/

^b^ https://www.blatchford.co.uk/endolite/silcare-breathe-locking-liner/

^c^ https://www.blatchford.co.uk/catalogue/liners/silcare-breathe-locking/ifu/938398_Iss1%20-%20Locking%20Liner.pdf.
